# From Innate Immunity to Metabolic Disorder: A Review of the NLRP3 Inflammasome in Diabetes Mellitus

**DOI:** 10.3390/jcm12186022

**Published:** 2023-09-17

**Authors:** Iris Maria Nițulescu, George Ciulei, Angela Cozma, Lucia Maria Procopciuc, Olga Hilda Orășan

**Affiliations:** 1 Department 4 of Internal Medicine, Faculty of Medicine, Iuliu Hatieganu University of Medicine and Pharmacy, 400012 Cluj-Napoca, Romania; nitulescu.iris@gmail.com (I.M.N.); angelacozma@yahoo.com (A.C.); olgaorasan@gmail.com (O.H.O.); 2 Department 2 of Molecular Sciences, Faculty of Medicine, Iuliu Hatieganu University of Medicine and Pharmacy, 400012 Cluj-Napoca, Romania; luciamariaprocopciuc@yahoo.com

**Keywords:** NRLP3 inflammasome, type 2 diabetes mellitus, interleukin-1β, insulin resistance

## Abstract

The role of the NLRP3 inflammasome is pivotal in the pathophysiology and progression of diabetes mellitus (DM), encompassing both type 1 (T1D), or type 2 (T2D). As part of the innate immune system, NLRP3 is also responsible for the chronic inflammation triggered by hyperglycemia. In both conditions, NLRP3 facilitates the release of interleukin-1β and interleukin-18. For T1D, NLRP3 perpetuates the autoimmune cascade, leading to the destruction of pancreatic islet cells. In T2D, its activation is associated with the presence of insulin resistance. NLRP3 activation is also instrumental for the presence of numerous complications associated with DM, microvascular and macrovascular. A considerable number of anti-diabetic drugs have demonstrated the ability to inhibit the NLRP3 inflammasome.

## 1. Introduction

The incidence of type 2 diabetes (T2D) has been on a steady rise, tripling in number over the last two decades. Worldwide, an estimated 463 million people between the ages of 20 and 79 had T2D in 2019 [1]. The causes of T2D are multifaceted, with factors such as genetics, diet (high in processed meats, refined carbs), and lack of physical activity playing a leading role [2]. These lead to an imbalance between the immune and metabolic system and to a state of low-grade inflammation. This contributes to the development of disorders such as obesity, diabetes, atherosclerosis, cardiovascular disease, gout, and neurodegenerative diseases [3].

Recent research on diabetic complications has focused on inflammasomes, particularly NLRP3. Inflammasomes are multi-protein scaffolding complexes which are part of the innate immune system. Once activated, inflammasomes lead to caspase-1 activation and release of interleukin-1β (IL-1β) and interleukin-18 (IL-18). One other significant effect is the initiation of gasdermin D-mediated pyroptosis, an inflammatory form of programmed cell death. IL-1β has been recognized to be central in the development of complications for diabetes mellitus (DM) [4].

This review will present the mechanisms of NLRP3 activation and its importance for the initiation of type 1 diabetes mellitus (T1D), T2D, and the subsequent chronic complications that arise. We also present the therapeutic implications of targeting the NLRP3 inflammasome for the management of DM.

## 2. Methods

For this review, PubMed, Scopus, Embase, and Web of Science were searched from May 2023 to September 2023 using the keywords diabetes mellitus, NLRP3, inflammasome, IL-1β, IL-18, and diabetes complications.

## 3. Innate Immunity and Disease—Complex Role of NLRP3 Inflammasome

The innate immune system identifies markers of microbial infections and cellular damage. Molecular structures like viral nuclei acids, flagellin, and bacterial lipopolysaccharides (LPS)—referred to as pathogen-associated molecular patterns (PAMPs)—activate pattern recognition receptors (PRRs) and trigger the innate immune system. Apart from PAMPs, PRRs also detect the release of molecules from dying cells, termed damage-associated molecular patterns (DAMPs). However, not all innate immune receptors are directly activated by PRRs. Instead, some of them respond to the cellular alterations induced by PAMPs or DAMPs [5].

Certain inflammasomes function as PRRs, while others detect cellular disturbances. NLRP3, also referred to as cryopyrin, detects cellular stress and cell membrane damage. It is currently the most extensively researched inflammasome. NLRP3 belongs to the NLR family and is distinguished by its nucleotide-binding domain (NBD) and leucine-rich repeats (LRR) [6]. Expression of NLRP3 has been observed in neutrophils, monocytes, dendritic cells, barrier cells, lymphocytes, and neurons [7]. 

**Figure 1:**
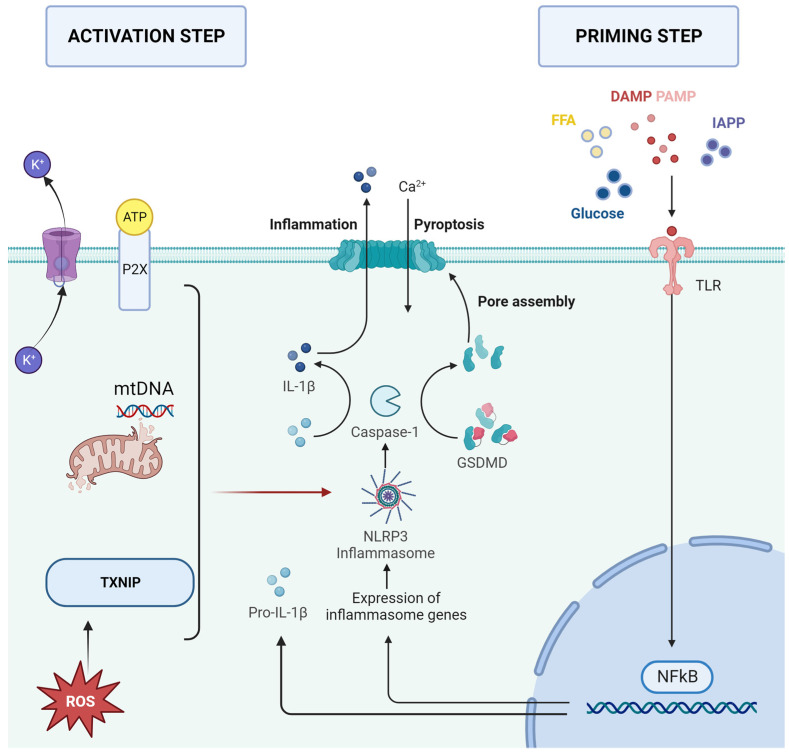
Factors that trigger the priming and activation of the NLRP3 inflammasome. DAMP—damage-associated molecular patterns; FFA—free fatty acids; GSDMD—gasdermin D; IAPP—islet amyloid polypeptide; IL-1β—interleukin-1β; IL-18—interleukin-18; mtDNA—mitochondrial DNA; PAMP—pathogen-associated molecular pattern; P2X—P2X purinergic receptor; ROS—reactive oxygen species; TLR—Toll-like receptor; TXNIP—thioredoxin interaction protein.

The activation of NLRP3 is a two-step process that requires first a priming process followed by an activation step (Figure 1). Priming can be triggered by Toll-like receptors (TLR) once they recognize DAMPs and PAMPs, free fatty acids (FFA), glucose, or islet amyloid polypeptide (IAPP). These initiate NF-κB-mediated upregulation of NLRP3, pro-IL-18, and pro-IL-1β. The activation and assembly of the inflammasome can be triggered by K^+^ efflux, Na^+^ influx, Cl^−^ reduction, intracellular Ca^2+^ overload, reactive oxygen species (ROS), the P2X purinergic receptor, mitochondrial DNA (mtDNA) damage, or thioredoxin interaction protein (TXNIP) [8].

The signaling pathway of the NLRP3 inflammasome encompasses three distinct elements: the sensor, adaptor, and effector. Respectively, these components are represented by NLRP3, ASC (apoptosis-associated speck-like protein with a caspase recruitment domain), and caspase-1. Upon activation of NLRP3, ASC recruits caspase-1 and cleaves it into its active form. The role of caspase-1 is to cleave gasdermin D (pyroptosis initiator) and also to cleave pro-cytokines into their active, proinflammatory form [5].

The role of NRLP3 in human pathology first came to light when several inherited (autosomal-dominant) autoinflammatory diseases were discovered to be a part of cryopyrin-associated periodic syndrome (CAPS). These mutations lead to the presence of three chronic, aseptic inflammatory diseases: familial cold autoinflammatory syndrome (the mildest form), Muckle–Wells syndrome (the intermediate form), and neonatal onset multi-systemic inflammatory disease/chronic infantile neurological cutaneous articular syndrome (the most severe form). These disorders manifest with recurrent episodes of inflammation that lead to fever, joint, skin, and ocular symptoms. In the most severe form, central nervous system inflammation symptoms are also present. The monocytes and macrophages isolated from these patients display a high level of IL-1β in the absence of any inflammatory stimulus [9].

## 4. The NLRP3 Inflammasome’s Impact on T1D Pathophysiology

The immune-mediated destruction of pancreatic β-cells in T1D is driven by T-cells that infiltrate the pancreatic islets. The NLRP3 inflammasome has been implicated in various autoimmune disorders such as rheumatoid arthritis, multiple sclerosis, and inflammatory bowel disease [10]. Recent research has deepened the understanding of the NLRP3 inflammasome in T2D, but there is growing evidence of its role in T1D pathophysiology as well [11]. 

The knockout of NLRP3 in mice was shown to inhibit T-cell activation and Th1-cell differentiation [12]. Furthermore, mitochondrial dysfunction can lead to the release of oxidized mtDNA, which in turn activates the NLRP3 inflammasome. The release of mtDNA increases the presence of T-cells in pancreatic lymph nodes, and this in turn promotes T1D. This effect is absent in mice with knockout for NRLP3 [13]. Exposing islet cells to LPS boosts the expression of NLRP3 and IL-1β [14]. IL-1β not only promotes movement of the inflammatory cells into the islets but also directly induces cytotoxic and apoptosis. Individuals with T1D, whether newly diagnosed or long-standing, display heightened serum IL-1β levels compared to healthy subjects. A reduced expression of IL-1 receptor antagonist (IL-1RA) leads to increased IL-1β production and β-cell dysfunction and pyroptosis [10]. Pancreatic islets of non-obese diabetic mice pretreated with IL-1RA exhibited downregulation of the NOS2a, COX2, IL-6, IL-1β, and HMOX1 genes, and maintained viability and function [15]. It is noteworthy that mice lacking caspase-1, despite having diminished IL-1 and IL-18 levels, demonstrated comparable diabetes incidence and streptozotocin sensitivity to regular mice [16]. Similarly, blocking IL-1 in another mouse model using either an anti-IL-1β antibody or IL-1 trap did not yield significant benefits [17]. Similarly, blocking IL-1 in another mouse model using either an anti-IL-1β antibody or IL-1 trap did not yield significant benefits [17] While IL-1β does play a central role in T1D pathogenesis, gene knockout can trigger compensatory mechanisms that upregulate genes which fulfil similar functions. In the case of IL-1β, upregulation of other cytokines (such as TNF-α or IL-12) would be one such mechanism. Several immune anomalies participate in T1D, but no single one is indispensable for its progression. Other gene knockouts, for IL-4, IL-10, and interferon-γ, have also not been able to prevent T1D [16]. 

While many studies suggest an upregulation of NLRP3 as a contributing factor to T1D onset, research has shown that T1D patients exhibit downregulation of NLRP3, NLRP1, caspase-1, and IL-1β in blood mononuclear cells and granulocytes [18]. Consequently, it remains uncertain whether NLRP3 downregulation is a cause or result of T1D progression. In systemic lupus erythematosus, NLRP3 is also downregulated, correlating inversely with disease severity [19]. Furthermore, enzymes besides caspase-1 can cleave pro-IL-1β into IL-1β. This has been observed in neutrophil- and macrophage-derived serine proteases such as proteinase-3 (PR3), elastase, and cathepsin-G [20]. 

SNPs in inflammasome genes have been linked with an increased risk of T1D. Notably, the SNPs rs11651270 and rs2670660, found in the NLRP1 inflammasome gene, were associated with T1D in a Chinese Han population. The minor allele C of rs11651270 was significantly associated with lower risk of T1D compared with the T allele (OR = 0.71, 95% CI 0.57–0.88). Patients with the genotype GG or GA of rs2670660 had a lower prevalence of T1D (OR = 0.71, 95% CI 0.54–0.93). Moreover, individuals with the TT genotype of rs11651270 exhibited a lower age of T1D onset [21]. As for NLRP3, the SNP rs10754558 showed an association with the risk of T1D in a Brazilian population. The G allele was protective against the development of T1D (OR = 0.65, 95% CI 0.48–0.88) [22].

## 5. Inflammasome Activation—Link between IL-1β, IL-18, and T2D

The NRLP3 inflammasome is important for the pathogenesis of insulin resistance and T2D. Aberrant glucose metabolism induces chronic inflammation through NRLP3. Hyperglycemia activates it by the ATP/P2X purinergic receptor 4 and the induction of TXNIP [23]. Islet cells exposed to chronic hyperglycemia will activate NLRP3 and induce IL-1β secretion [8]. This in turn will promote insulin resistance. Other activators of NLRP3 in islet cells are saturated fatty acids and islet amyloid polypeptides [24,25].

Chronic elevation of IL-1β leads to an increase in insulin levels, which enhances glucose uptake and metabolism in macrophages, upregulates insulin receptor expression, and amplifies their inflammatory status. IL-1β alone increases glucose uptake in macrophages [11]. Inflammasome components and IL-1β are prominently expressed in human adipose tissue, predominantly in macrophages, and are linked to insulin resistance. Visceral adipose tissue in overweight or obese individuals shows heightened NLRP3 expression compared to their subcutaneous tissue. Elevated levels of glucose, saturated free fatty acids, and uric acid stimulate NLRP3 and lead to release of IL-1β and other cytokines. Suppression of NLRP3 enhances insulin signaling across adipose, liver, and skeletal muscle tissues [11]. In contrast, NLRP3 knockout mice exhibit better glucose tolerance and insulin sensitivity, alongside decreased levels of circulating IL-18 and reduced signs of IL-1β and caspase-1 activation in adipose tissue [26,27]. 

As for IL-18, individuals with T2D or impaired glucose tolerance have elevated serum levels compared to healthy individuals. The A/A genotype of the IL-18 gene promoter’s -607 C/A polymorphism has been linked to a higher incidence of T2D [28]. Regarding NLRP3 polymorphisms, SNP rs10754558 was verified for its role in T2D risk in a Chinese population in two studies. In the first study, the GG genotype led to a higher risk of T2D (OR = 1.25, 95% CI 1.06–1.74). Patients with the GG genotype also had higher LDL-C, fasting insulin, and HOMA-IR values [29]. The G allele leads to an increased quantity (1.3-fold) of NLRP3 mRNA when compared to the C allele [30]. In the second study, again the GG polymorphism was associated with T2D (OR = 1.81, 95% CI 1.16–2.83) [31]. In one diet intervention study, patients were randomized to a low-fat or a Mediterranean diet for three years. Non-diabetic patients with the CT or TT genotype of the rs4612666 SNP and AG or AA carriers of the rs10733113 SNP had an increased insulin sensitivity index [32]. Another NLR3P polymorphism that leads to an overactive inflammasome with overproduction of IL-1β is rs35829419. Patients with the A allele had a higher risk of T2D (CA+AA versus CC with OR = 2.05, 95% CI = 1.16–3.75) [33]. Other significant SNPs associated with increased risk of T2D were rs10925027 (CC genotype) rs4925659 (GG genotype) and rs10754558 (GC and GG genotypes) [34].

## 6. NLRP3: At the Heart of Diabetic Cardiovascular Concerns

DM is a leading factor for cardiovascular disease, elevating the chances of stroke or heart attack (major cardiovascular events—MACE) by two to three times. This heightened risk stems primarily from an increased prevalence of arterial hypertension and accelerated atherosclerosis. Effectively managing diabetic cardiovascular disease (DCD) necessitates the control of blood pressure, lipids, and blood sugar levels. Certain anti-diabetic medications, specifically, GLP-1 receptor agonists and SGLT2 inhibitors, offer added cardioprotective benefits beyond their primary role in lowering blood sugar [4].

The involvement of NLRP3 in DCD is underscored by studies showing that inhibition of IL-1β reduces the incidence of MACE. The Canakinumab Anti-Inflammatory Thrombosis Outcomes Study (CANTOS) demonstrated that canakinumab, an IL-1β inhibitor, effectively lowered MACE rates in patients with or without diabetes. Indicators of systemic inflammation, such as serum hsCRP and IL-6, were also reduced. This protective effect was achieved independently of low-density cholesterol levels (LDL-C) [35]. Studies in mouse models of atherosclerosis revealed that a deficiency or antagonism of IL-1β or IL-18 lessened the severity of atherosclerotic lesions [36,37]. In another rat model of T2D, NLRP3 silencing mitigated adverse cardiovascular changes [37,38]. Increased risk for macrovascular complications was observed in patients with the NLRP3 polymorphism rs35829419 (for the A allele in males, OR = 4.44, 95% CI 1.39–14.25) [39].

Studies utilizing cellular or animal models have demonstrated that, in the presence of hyperglycemia, NLRP3 induces endothelial inflammation. Both IL-1 antagonism and NLRP3 knockdown have been shown to hinder the formation of adhesion molecules within atherosclerotic plaques. For individuals with DM, a positive correlation exists between serum IL-1β levels and carotid atherosclerosis [40]. Additionally, oxidized LDLs act as NLRP3 activators [41]. In a pig model examining atherosclerosis and DM, increased levels of sterol regulatory element binding protein (SREBP)-1 in the aorta corresponded with an upregulation in NLRP3 expression [42]. Cholesterol crystals are primarily implicated in triggering NLRP3 activation in atherosclerosis [43]. The NLRP3 inhibitor, MCC950, diminished atherosclerotic progression in a mouse model by decreasing oxidative stress and dampening the expression of inflammatory genes, notably reducing the secretion of Il-1β and caspase-1 [44]. 

Cardiac macrophage production of IL-1β play has been implicated in increasing arrhythmia risks in diabetic mice [38]. The NLRP3 inflammasome is elevated in atrial myocytes in patients with atrial fibrillation (AF) (be it paroxysmal or long-standing). Mice with knock-in to express NLRP3 showed a heightened predisposition to AF [45]. Using the NLRP3 inhibitor MCC950 or genetic knockout of NLRP3 on mice led to a decreased incidence of AF. NLRP3 can cause a reduction in the effective refractory period by amplifying the ultra-rapid delayed rectifier K^+^ current and the acetylcholine-regulated K^+^ current [46].

Studies have examined the influence of exercise on the modulation of NLRP3 expression and its ramifications for cardiovascular health. The intensity of the aerobic exercise can modulate the body’s inflammatory response. High-intensity aerobic exercise, where 90% of the maximum heart rate is maintained for 40 min, leads to an increase in NLRP3 mRNA expression in peripheral mononuclear cells, accompanied by elevated IL-1β and IL-18 serum levels. Moderate intensity exercise, with maximum heart rate maintained at 70% for 40 min, has an inverse effect [47]. This difference has been explained in part by the presence of significant oxidative stress that accompanies high-intensity effort [48]. An 8-week high-intensity exercise regimen (starting from 70% of the maximal oxygen uptake and upwards) has been observed to reduce IL-18 mRNA in the adipose tissue of obese individuals [49]. When comparing aerobic and resistance training, aerobic exercise is better at lowering TNF-α, IL-1β, and IL-18 levels in adipose tissue [50]. In obese mice fed a high-fat diet (HFD) there was an upswing in the expression of NLRP3, caspase-1, and IL-1β within the myocardium. These alterations reverted when the mice engaged in running over a span of 12–14 weeks [51]. On its own, an HFD can induce a DCD phenotype in mice and compromised diastolic function. At the same time, NLRP3 formation increases in the left ventricle and negatively correlates with the ejection fraction. Treadmill exercising in this case leads to lower NLRP3 expression [52]. The American Diabetes Association recommends that patients with DM participate in aerobic and resistance exercise consisting of more than 150 min/week, spread over 3–5 days [53]. 

## 7. Inflammatory Mechanisms in the Onset of Diabetic Kidney Disease

Diabetic kidney disease (DKD) is the primary cause of chronic kidney disease in 30 to 40% of patients diagnosed with DM [54]. As with DCD, the management of DKD encompasses hypertension treatment, glycemic regulation, and weight management. The emergence of DKD has been linked to factors such as hypertension, activation of the renin–angiotensin system, hyperglycemia, and dyslipidemia. However, present research is also showing the important influence of immune and inflammatory mechanism. Leukocytes are the presumed source of inflammatory proteins, and the inhibition of their recruitment in the kidney tissue is protective against DKD [4]. Both serum and urinary levels of IL-1β and IL-18 are notably elevated in patients with DKD. While infiltrating monocytes are identified as the primary producers of IL-1β, kidney cells also express IL-18, particularly during episodes of acute injury [55]. 

In experimental models of DM, there was an upregulated glomerular expression of IL-1β, IL-18, and NLRP3. Notably, these alterations emerged before the manifestation of hallmark abnormalities seen in DKD, such as albuminuria and extracellular matrix accumulation. This implies that NLRP3 plays a pivotal role in the onset of DKD. Reinforcing this notion, the development of DKD was significantly attenuated when deficiencies in NLRP3 or caspase-1 were introduced in mouse models of DM [56,57]. 

## 8. Inflammatory Mechanisms and NLRP3 in Diabetic Retinopathy

Diabetic retinopathy (DR) is a common DM complication and a leading cause of visual impairment. Approximately 35% of individuals with DM will encounter DR, with the severity intensifying with age and the duration of the disease. There are two main stages of DR: non-proliferative DR, characterized by microaneurysms and retinal hemorrhages, and proliferative DR, marked by preretinal vascularization. Microvascular, genetic, and inflammatory factors contribute to the development of DR. Central to this are macrophages, which consistently release both inflammatory and angiogenic cytokines. These cytokines eventually instigate fibrosis and compromise the integrity of the blood–retinal barrier. Subsequent fluid leakage into the neural retina gives rise to exudates, leading eventually to macular edema [4].

Hyperglycemia and hypertension are primary risk factors for DR. Prolonged hyperglycemia causes upregulation of TXNIP, a modulator of NLRP3, which in turn will activate inflammatory signaling pathways. As neutrophils and monocytes accumulate in the retina, they release IL-6, TNF-α, IL-1β, and IL-18 [4]. With the aggregation of neutrophils and monocytes in the retina, there is a release of inflammatory cytokines like IL-6, TNF-α, IL-1β, and IL-18. Analogous to the patterns in DKD, there is an early upregulation of NLRP3, augmented levels of IL-1β and caspase-1, and electroretinographic anomalies before the onset of discernible microvascular complications [58]. 

MCC950 when applied to human retinal endothelial cells counteracted the dysfunction and apoptosis typically induced by high glucose levels [59]. Patients with proliferative DR have a higher expression of NLRP3, caspase 1, and IL-1β in proliferative membranes. They also exhibit elevated levels of IL-1β and IL-18 in the vitreous humor compared to individuals with non-proliferative DR [59,60]. Prostaglandins are also responsible for activation of NLRP3 and caspase-1. When rats were administered AH6809, a prostaglandin E2 antagonist, there was a marked decrease in inflammation indicators [61]. Diabetic mice with a deficiency in NLRP1 displayed suppressed inflammation and exhibited mitigation of retinal abnormalities [62].

## 9. Influence of NLRP3 on NAFLD in Diabetic Patients

Nonalcoholic fatty liver disease (NAFLD) is prevalent in about a quarter of the global population and up to 75% of those diagnosed with T2D. It is characterized by the accumulation of lipid droplets in more than 5% of hepatocytes, once other causes such as alcoholism or viral infections have been excluded. The severity of NAFLD varies, spanning from hepatic steatosis to cirrhosis. Hepatic steatosis is also closely linked with insulin resistance and metabolic syndrome, since insulin resistance increases hepatic uptake and synthesis of free fatty acids [63]. 

NAFLD incidence is not only higher in patients with DM, but they also have face an up to three times higher risk of progression towards end-stage liver disease and liver cirrhosis [64]. Multiple factors favor the transition from hepatic steatosis to non-alcoholic steatohepatitis (NASH). These include low-grade chronic inflammation, increased liver uptake of free fatty acids, liver glucose metabolism alterations, oxidative stress, accumulation of advanced lipoxidation end products, IL-6 and TNFα released by adipose tissue, and endotoxins from the gut microbiota [4]. Patients with NASH have heightened expression levels of NRLP3 and its associated cytokines, such as pro-IL-1β and pro-IL-18, when compared to those who only have steatosis [65]. Various stimuli, including ROS, free fatty acids, cholesterol crystals, and advanced lipoxidation or glycation end products, can activate NLRP3 [4].

In a mouse model, quercetin and allopurinol alleviated liver inflammation and steatosis induced by hyperglycemia. These beneficial effects were attributed to their antioxidative properties, which inhibited the ROS/TXNIP/NLRP3 pathway. Despite these results, NLRP3 and NLRP6 genetic deficiency was found to lead to intestinal dysbiosis. This led to subsequent inflammation, which in turn led to a worsening of the metabolic syndrome [66]. 

## 10. NLRP3 and Diabetic Neural Challenges 

Diabetic peripheral neuropathy (DPN) stands as a prominent microvascular complication, affecting approximately 29% of individuals with diabetes mellitus (DM). The pathogenesis of DPN involves peripheral nerve injury, reduced nerve conduction velocity, altered nerve fiber Na^+^, K^+^-ATPase activity, and the release of ROS. Hyperglycemia is the main cause of these changes. DPN compromises both motor and sensory neurons, resulting in pain in the lower limbs and increasing the likelihood of foot ulcers [67,68]. The function of peripheral nerves relies on the numerous Schwann cells that surround the axon. Schwann cells are sensitive to glucose and insulin fluctuations, with hyperglycemia being capable of instigating cell apoptosis [69]. Inhibition of glycemia-induced ROS formation in Schwann cells prevents NLRP3 activation and pyroptosis [70].

Cognitive dysfunction, manifesting as impairments in learning, memory, and executive functioning, has been recognized as an additional complication of DM. The etiology is again multifaceted and involves endothelial cell dysfunction, inflammation, and oxidative stress. This cognitive dysfunction is correlated with elevated inflammation markers such as hs-CRP, Il-6, and TNF-α [71]. Research on rat models of DM has unveiled a notable association between NLRP3 activation and cognitive deficits. Furthermore, when this activation was inhibited, a significant reduction in the expression of IL-1β, NLRP3, and caspase-1 within the hippocampus was observed [72,73]. 

## 11. Impact of Diabetic Medications on NLRP3 Modulation

An array of established anti-diabetic medications have shown a significant effect against the involvement of NRLP3 in diabetic complications. 

Metformin is one of the most used drugs in T2D. It inhibits caspase-1 and the formation of IL-1β and as such it also lowers insulin resistance [74]. Metformin can also modulate the AMPK/TOR signaling pathway in DCD [75] and has shown it can protect against myocardial ischemia–reperfusion injury and cell pyroptosis [76]. 

Dapagliflozin, an SGLT-2 inhibitor, in a mouse model, has been shown to significantly decrease the activation of the NLRP3 inflammasome in DCD. It improved the left ventricular end-systolic and end-diastolic volumes, and also the left ventricular ejection fraction. Its effect was dependent on activating AMPK/TOR pathway. The combination of dapagliflozin and ticagrelor had an additive effect [77]. Empagliflozin attenuates heart failure in experimental models of heart failure without DM by lowering intracellular Ca^2+^ and suppressing NLRP3 [78]. 

Regarding GLP-1 receptor agonists, both liraglutide and exenatide have shown protective outcomes. These agents not only mitigate neurological damage but also obstruct the onset of non-alcoholic fatty liver disease (NAFLD) and cardiomyocyte pyroptosis in diabetic rats. Their mechanism of action revolves around the inhibition of NLRP3 inflammasome activation [79,80,81,82]. Exenadin-4, a related compound, offers similar benefits [83]. 

Saxagliptin (a DPP4 inhibitor) has been observed to diminish NLRP3 inflammasome activation, subsequently decelerating the advancement of DCD [84].

Pioglitazone, a thiazolidinedione anti-diabetic drug, reduces ROS releases and downregulates NF-κB and thereby inhibits NLRP3. This has been shown to alleviate glomerular damage from DKD [85]. 

Acarbose, an α-glucosidase inhibitor, improved vascular endothelial dysfunction in the aorta of rats with T2D. By blocking NOX4-dependent superoxide generation, it also prevented NLRP3 activation [86]. Reducing oxidative stress and inhibition of NLRP3 has also been observed with the insulin secretagogue glibenclamide [87].

Glyburide, a sulfonylurea drug, is another drug utilized to treat T2D. It acts by inhibiting ATP-sensitive potassium channels in pancreatic β-cells. Its inhibitory action is specific to the NLRP3 inflammasome, since glyburide does not impact the release of IL-1β from other pathways such as NLRC4 or NLRP1. While glyburide has shown inhibitory effects both in vitro and in vivo against NLRP3 activation, its high doses in vivo can lead to hypoglycemia, limiting its use for T2D treatment [88]. 

Regarding non-diabetic medications, rosuvastatin, a statin drug, improved diastolic dysfunction and cardiac fibrosis in a rat model of DCD. The effects were dependent on the inhibition of NLRP3 inflammasome via TXNIP [89]. Melatonin, a hormone used for sleep regulation, also has anti-inflammatory potential. In diabetic mice, melatonin was found to mitigate endothelial cell pyroptosis, slow the progression of atherosclerosis, and suppress the pyroptosis of both neurons and cardiomyocytes. These effects were mediated through the regulation of the NLRP3 axis [90,91]. 

Verapamil is a calcium channel blocker used for treating arterial hypertension and angina pectoris. It also improves insulin resistance. Verapamil inhibits the assembly of NLRP3 in mice with DR, reduces the release of IL-1β in the vitreous humor, and inhibits pathological angiogenesis [92]. In rodent studies, both the antibiotic minocycline and 1,25-dihydroxyvitamin D exhibited positive outcomes by obstructing the ROS/TXNIP/NLRP3 pathway [93,94]. Fenofibrate, a triglyceride-lowering drug, has also shown potential benefits for DR. It has the ability to mitigate inflammation, vascular leakage, and the progression of DR, with NLRP3 inhibition being one of the underlying mechanisms driving these off-label effects [95,96]. 

## 12. Directions for Future Research

The NLRP3 inflammasome’s role in diabetes remains a topic of profound interest for research. While numerous animal models studies have proven the molecular mechanism that links NLRP3 and T1D or T2D, the issue remains in replicating and showing the same benefit for human subjects. A wide range of anti-diabetic medications have proven inhibition against NLRP3, yet DM microvascular and macrovascular complications remain a challenge. It is to be seen if a more targeted approach against the NLRP3 pathway can provide additional benefit, or if it can be achieved without significant impact on the innate immune system. Additionally, genetics of the NLRP3 inflammasome will be integrated so as to find individuals at risk for T1D, T2D, or that can respond better to a particular treatment.

Current treatment agents for NLRP3-related diseases include drugs that target IL-1β: anakinra, canakinumab, and rilonacept. The concern is that these drugs also increase the risk of infection. Of the direct NLRP3 inhibitors (MCC950, tranilast, OLT1177, INF39, oridonin), tranilast is the most studied in humans. It has proven beneficial effects for patients with early DKD while having a favorable safety profile. At this point, direct inhibitors have not been approved for use in clinical trials or received recommendation by medical institutions [97]. 

## 13. Conclusions

The role of the NLRP3 inflammasome in the onset and progression of DM has become increasingly clear in recent years. As a key regulator of immunity and inflammation, it plays a pivotal role in the pathogenesis of both T1D and T2D. Almost every major complication of DM, affecting various organ systems including cardiovascular, neurological, renal, and hepatic, is associated with the upregulation of NLRP3. Numerous diabetic medications target the NLRP3 inflammasome, offering therapeutic advantages beyond their primary glucose-lowering effects.
